# Cisplatin-Mediated IL-6 and IDO1 Suppression in Mesenchymal Stromal Cells: Implications for Tumor Microenvironment Modulation In Vitro

**DOI:** 10.3390/cimb47040231

**Published:** 2025-03-27

**Authors:** Armin von Fournier, Erik Würflein, Helena Moratin, Manuel Stöth, Totta Ehret Kasemo, Marietta Herrmann, Miguel Goncalves, Rudolf Hagen, Stephan Hackenberg, Thomas Gehrke, Agmal Scherzad

**Affiliations:** 1 Department of Oto-Rhino-Laryngology, Head and Neck Surgery, University Hospital Wuerzburg, 97080 Wuerzburg, Germany; 2 Department of Oral and Maxillofacial Surgery, Albert-Ludwigs University Freiburg, 79106 Freiburg, Germany; 3 IZKF Group Tissue Regeneration in Musculoskeletal Diseases, University Hospital Wuerzburg, 97074 Wuerzburg, Germany; 4 Bernhard-Heine-Centrum for Locomotion Research, Julius-Maximilians-Universitat Wuerzburg, 97074 Wuerzburg, Germany

**Keywords:** cisplatin, MSC, immunomodulation, tumor microenvironment, IL-6, IDO1

## Abstract

Mesenchymal stromal cells (MSCs) influence tumor biology and immunology by releasing cytokines, chemokines and growth factors. Currently, cisplatin is an integral part of drug-based tumor therapy, for example, in head and neck squamous cell carcinoma (HNSCC). Cisplatin treatment induces apoptosis as a primary mechanism of action; however, additional immunomodulatory effects of cisplatin are gaining interest. The aim of this study is to evaluate the possible immunomodulatory effects of cisplatin in human MSCs (hMSCs). The MSCs, obtained from human bone marrow, were characterized by analyzing plastic adherence, typical surface features, and ability to differentiate. Toxicity analysis of cisplatin’s effects on primary MSCs, including the determination of a subtoxic concentration, was performed using the MTT assay. Enzyme-linked immunosorbent assays (ELISA) and a quantitative real-time polymerase chain reaction (qRT-PCR) were used to identify potentially immunomodulatory factors. Additionally, a scratch assay was performed to evaluate cell migration. First, subtoxic cisplatin concentrations were determined. A significantly reduced protein expression of indoleamine 2,3-dioxygenase 1 (IDO1) in MSCs under the influence of subtoxic cisplatin concentrations was demonstrated. Similarly, IL-6 protein expression was qualitatively reduced at subtoxic concentrations, although without statistical significance. At the mRNA level, qRT-PCR showed a non-significant, cisplatin concentration-dependent reduction in the expression of both IL-6 and IDO1. The scratch assay showed no statistically significant influence on migration after cisplatin treatment. In MSCs, there is tendency to a decrease in IL-6 and IDO1 at both protein and mRNA level after cisplatin exposure. These effects are congruent with each other and dose-dependent. This indicates that cisplatin not only acts via the known cytotoxic effect, but may induce a reduction in tumor-supporting proteins, like IL-6 and IDO1, by MSCs in the tumor microenvironment at subtoxic concentrations. Traditional cytostatic compounds, which can favorably modulate the immune system in the tumor microenvironment, may open new avenues to explore treatment strategies specifically targeting immunomodulation. Overall, the results indicate beneficial immunomodulation by cisplatin.

## 1. Introduction

Mesenchymal stromal cells (MSCs) are the most common designation for multipotent progenitor cells, and they can be isolated from various tissue types, such as cartilage, bone marrow or adipose tissue [1]. A key function is their ability to differentiate into different functional cell types under specific conditions. Specifically, their osteogenic, chondrogenic, and adipogenic differentiation can be precisely controlled under in vitro conditions [2]. Furthermore, MSCs have immunomodulatory properties that can influence both the innate and the acquired immune system. In addition to direct cell–cell contact, immunomodulation by MSCs is also mediated by paracrine stimuli. This property of MSCs is utilized, for example, in the treatment of graft-versus-host disease. This therapeutic application has already been used in preclinical studies, with promising results. Moreover, there is intensive research activity focused on the use of MSCs in the field of tissue engineering to generate or modify specific tissues [3,4]. For example, haematopoietically active components, such as those derived from the bone marrow, can be transferred to a patient with bone marrow damage resulting from radiation or chemotherapy [5,6,7,8]. In the present study, references to MSCs are, for simplicity, used interchangeably for bone marrow-derived MSCs and MSCs of other origins.

Physiologically, protein secretion is crucial for processes like the formation of the extracellular matrix, as well as the immunomodulatory properties of MSCs. Immunomodulatory properties are also of great importance in malignant tumor diseases, especially within the tumor microenvironment (TME), where MSCs influence tumor biology and immunology by releasing cytokines, chemokines and growth factors [1,9,10] such as transforming growth factor-β1 (TGF- β1), tumor necrosis factor-α (TNF-α), interferon-gamma (IFN-γ) and hepatocyte growth factor (HGF), as well as interleukin-6 (IL-6) and indoleamine 2,3-dioxygenase (IDO) [11,12,13].

A pleiotropic but immunosuppressive function has been observed for IL-6 secretion, from which malignant tumors benefit by enhancing proliferation, survival, invasiveness and metastasis [2,3]. Furthermore, the immunosuppressive enzyme IDO is secreted by human MSCs. Several unfavorable effects of IDO have already been demonstrated in tumor immunology and the tumor microenvironment, as well as specifically in tumor therapy with cisplatin [14,15,16,17]. It has been shown that IDO inhibits the proliferation of CD8(+) T cells and B cells in vitro and promotes tumor growth [18].

Cis-diaminedichloroplatinum (II) (cisplatin) is one of the most important chemotherapeutic agents in the treatment of numerous malignant tumors in humans, such as head and neck, lung, bladder, ovarian, testicular carcinoma or other tumor entities such as germ cell tumors, sarcomas and lymphoma. The primary mechanism of action is apoptosis-induction through DNA cross-link formation and mitosis inhibition [19]. Studies conducted in recent years suggest that cisplatin also has a relevant immunomodulatory effect. De Biasi et al. (2014) reviewed anti-tumor immunomodulation caused by cisplatin and described four main mechanisms: (I) upregulation of the MHC class I complex, (II) proliferation and recruitment of effector cells, (III) upregulation of the lytic activity of cytotoxic effectors and (IV) downregulation of the immunosuppressive microenvironment [20]. The function of MSCs in the course of the peritumoral biological processes also falls under this category. It has been suggested that cisplatin may achieve anti-tumor activity via its ability to mediate immunomodulation, as chronic cisplatin exposure promotes the intratumoral accumulation of T cells. Interestingly, MSC-associated antitumor effects decreased after induction by cisplatin [21]. Another study demonstrated a beneficial modulation of the immune environment, yet the authors emphasized that deeper knowledge of chemotherapy-induced immune modulation is clearly required [22]. The specific impairment of MSCs by cisplatin still remains unclear.

The current study hypothesizes that cisplatin influences the immunomodulatory functions of MSCs via suppression of the tumor promotors IL-6 and IDO. The hypothesized clinical relevance is that cisplatin-induced reduction in MSC-derived IL-6, IDO or both could support cancer therapy by removing immunosuppressive factors from the tumor microenvironment. Given the contradictory data in the literature on the immunomodulatory effects of cisplatin, this study aims to clarify these interactions in vitro.

## 2. Materials and Methods

MSCs from residual acetabular reaming material from patients receiving total hip arthroplasty were used. Donor consent from the eight donors was obtained prior to surgery, and experiments with donor cells were approved by the ethics committee of the Julius Maximilian University of Würzburg, Germany (91/19-me).

### 2.1. Preparation of the Cells

The spongiosa was transferred to a 50 mL centrifuge tube (GB227270-N, Greiner Bio-One GmbH, Frickenhausen, Germany) and filled with 50 mL of a mixture of equal parts Dulbecco’s modified Eagle’s medium (DMEM) and DMEM: Nutrient Mixture F-12 (DMEM/F12). The tube was shaken by hand to detach the cells from the spongiosa and centrifuged at 1200 rpm for 5 min at RT (centrifuge 5810 R Eppendorf AG, Hamburg, Germany).

After removing cell culture medium and fat (membrane vacuum pump, KNF Neuberger GmbH, Freiburg, Germany), 50 mL of DMEM/F12 were added. After shaking and sedimentation of the remains of bone and connective tissue, the cell-rich medium was transferred to a new tube. The final supernatant containing cells was centrifuged at 1200 rpm for 5 min. The supernatant was discarded, and the remaining cell pellet was resuspended in stromal cell expansion medium (DMEM-EM; DMEM, 10% heat-inactivated fetal calf serum [FCS, BSF, Linaris, Wertheim, Germany], 1% penicillin/streptomycin [Biochrom AG, Berlin, Germany]). The cell number was determined in the hematocytometer. At least 8 × 10^8^ to ideally 1 × 10^9^ cells were seeded in 175 cm^2^ cell culture flasks (GB660160, Greiner Bio-One GmbH) with 27 mL DMEM-EM. Three to four days later, cells were washed with 15 mL of sterile phosphate-buffered saline (PBS, Roche Diagnostics, Mannheim, Germany) and then refilled with 21 mL fresh medium. After trypsinization and reseeding, the cells were used for the experiments. Cells were used in passages 1–3.

To determine the number of cells and cell viability by means of triple measurement with the cell counter CASY^®^ (Innovatis, Reutlingen, Germany), 10 mL of CASYton (isotonic saline solution, Innovatis, Reutlingen, Germany) was placed in a cell counter vessel (CASY-Cups Innovatis, Reutlingen, Germany). Also, 10 μL of cell suspension prepared by previously trypsinizing the cell culture was added and mixed.

The cells were cultured and at about 80% confluency, the cells were passaged by aspirating the medium and washing the cells with 10 mL of sterile PBS. Then, 5 mL of trypsin 0.25% EDTA 1× (Life Technologies Corp. [Gibco], Carlsbad, CA, USA) was added after warming to 37 °C in a water bath. The bottle was incubated in an incubator for 5 min and the enzymatic reaction was stopped by adding 5 mL DMEM-EM. Thereafter, the cells were detached from the plastic surface, resuspended in the medium and, after counting, kept in the culture or distributed on well plates as required. In total, cells from eight donors (*n* = 8) were used for the experiments. These were assigned the numbers 1 to 8.

### 2.2. Evidence of Eligible MSC Classification

To demonstrate that the criteria for identification of mesenchymal stromal cells according to the “Mesenchymal and Tissue Stem Cell Committee of the International Society for Cellular Therapy” were met, the adherence of the cells, differentiability by induction and histological staining and specific surface characteristics by flow cytometry (see below) were assessed [23].

### 2.3. Stromal Cell Differentiation and Histological Staining

Different types of differentiation were investigated exemplarily for donors 3 and 6 (*n* = 2) using the two following published protocols: (1) osteogenic differentiation and (2) adipogenic differentiation. Stromal cell differentiation was induced with differentiation additive added DMEM-EM. Cells were seeded in a flask incubated for 24 h (h). This was followed by fixation with 4% paraformaldehyde (PFA) in PBS for staining and histological analysis.

To induce osteogenic differentiation (over 21 days), a medium consisting of DMEM-EM with addition of 50 μg/mL L-ascorbic acid 2-phosphate, 10 mM β-glycerophosphate and 100 nM dexamethasone was used.

The adipogenic differentiation took place over 14 days. The medium consisted of DMEM-EM with the addition of 1 uM dexamethasone, 500 μM 3-isobutyl-1-methylxanthine (IBMX), 1 ug/mL insulin and 100 uM indomethacin.

Histological staining was performed after washing with distilled water and rehydration.

For staining according to von Kossa to demonstrate osteogenic differentiation through calcium detection, the covering was carried out with a 1% silver nitrate solution (Sigma-Aldrich Chemie GmbH, Steinheim, Germany) and irradiation with a UV lamp (Desaga Uvis, Desaga GmbH, Heidelberg, Germany) for 20 min. After washing, the addition of 5% sodium thiosulfate solution [Merck KGaA, Darmstadt, Germany] and washing again, the cell nuclei were stained with nuclear fast red solution [1001210500, Sigma-Aldrich Chemie GmbH, Steinheim, Germany]. After washing with distilled water, the cells were dried with ethanol.

Subsequently, a further calcium staining of the cells was carried out after washing with Alizarin Red S solution [TMS-008-C, Merck KGaA, Darmstadt, Germany]. In this process, water-insoluble red–orange salts are formed by reaction with calcium deposits in the cells.

With the Oil Red O stain, fat vacuoles were detected as a sign of adipogenic differentiation. After washing, the treatment was carried out with 100% 1,2-propanediol (propylene glycol, P4347-500ML, Sigma-Aldrich Chemie GmbH, Steinheim, Germany) for 5 min. After suction, the cells were incubated with Oil Red O [Sigma-Aldrich Chemie GmbH], previously heated to 60 °C and incubated at 60 °C for 10 min. Afterwards, the cells were treated with 85% 1,2-propanediol for 1 min. The cells were then washed with distilled water, which was repeated three times. Finally, the cell nuclei were stained with hematoxylin solution (1051740500, Merck KGaA, Darmstadt, Germany) for 30 s.

### 2.4. Flow Cytometry and Surface Characterization

Flow cytometry was used to examine MSCs with regard to their characteristically expressed surface antigens.

MSCs of donors 4, 5 and 6 (*n* = 3) were examined for a combination of stromal cell-typical markers CD73, CD90 and CD105 and absence of hematopoietic markers CD31, CD34 and CD45.

For this purpose, after MSCs had been detached and counted in the CASY, 2 × 10^5^ cells in DMEM were transferred to a FACS tube (BD 352002, Becton Dickinson GmbH, Heidelberg, Germany), centrifuged at 1400 rpm 5 min at RT and the supernatant was discarded. An amount of 1 mL PBS with 10% FCS was added to the cell pellet. After resuspension, cells were incubated on ice for 60 min. After centrifugation and decantation, antibodies were added according to the manufacturer’s instructions. After preparation, the samples were analyzed and displayed using the FACSdiva program (BD Bioscience, Heidelberg, Germany). The antibodies are listed in Table 1.

### 2.5. Cytotoxicity Evaluation with the 3-[4,5 Dimethylthiazol-2-yl]-2,5-diphenyltetrazolium-bromid (MTT) Assay

With this colorimetric method, cell activity was determined as an indicator of cell viability. To determine the cytotoxicity of cisplatin on MSCs, assays were carried out with increasing concentrations of cisplatin (Cisplatin Teva^®^ 1 mg/mL, 3.3 mM, Teva Pharmaceutical Industries Limited, Petach Tikwa, IL, USA). The cisplatin solution was further diluted with DMEM-EM. The concentrations 0.1 μM, 0.5 μM, 1 μM, 2 μM, 5 μM, 10 μM, 20 μM and 100 μM and a negative sample with DMEM-EM were used. After the addition of cisplatin and incubation for 24 h, washing with PBS and renewed incubation for 24 h was performed. The experiment was carried out three times. After aspirating the medium, MTT solution (1 mg/mL MTT, M5655-100MG, Sigma-Aldrich Chemie GmbH, Steinheim, Germany) was applied with DMEM-EM. Cells were incubated, the solution was aspirated and replaced with 850 μL/well 70% isopropanol (VWR Chemicals, Fontenay-sous-Bois, France). After 30 min incubation at RT in the dark, isopropanol was thoroughly resuspended and 100 μL were transferred to each well in a 96-well plate (6 technical replicates per 850 μL from original cell culture well) and analyzed at 570 nm in an ELISA microplate reader (ELx800 BioTek Instruments GmbH, Bad Friedrichshall, Germany). The result was imported into Excel (Excel 2016, Microsoft, Redmond, WA, USA) and the relative viability based on the negative control without cisplatin was calculated. Additionally, 1 mM cisplatin and 200 µM concentration tert-butyl hydroperoxide (tBHP) treatment for the same duration as cisplatin was used as the positive control.

After estimating the subtoxic range in preliminary measurements (*n* = 4), additional MTT assays with concentrations of 5 μM, 10 μM and 20 μM cisplatin were carried out with the cells of donors 1 to 8 (*n* = 8) to generate replicates for further experiments.

### 2.6. Migration Analysis with the Scratch Assay

Cells from all donors (*n* = 8) were used for this experiment. After transferring 5 × 10^5^ cells/well into a 12-well plate, treatment with 5 μM, 10 μM and 20 μM cisplatin and negative controls (DMEM-EM) were carried out. A vertical “scratch” was introduced in each well: a sterile 1 mL pipette tip was used to scratch an artificial “wound” (cell-free area) at the center of each well. After scratching, detached cells were removed by resuspending and aspirating the medium. Then, 1 mL/well PBS was added and microscopic images at 4× magnification were recorded (automated, inverse transmitted light microscope LEICA DMI 4000 B Leica Microsystems GmbH, Wetzlar, Germany; microscope control box LEICA CTR 4000, Leica Microsystems GmbH, Wetzlar, Germany; software LAS V4.8, Leica Microsystems GmbH, Wetzlar, Germany). PBS was then aspirated, 1 mL DMEM-EM was added and cells were incubated at 37 °C for 24 h and the imaging procedure was repeated after 24 h. Image analysis of “wound closure” was carried out with ImageJ (1.53k, Rasband, WS, USA. National Institutes of Health, Bethesda, MD, USA) [24], reporting a measure of the cell-free area for each image.

### 2.7. Protein Determination with Enzyme-Linked Immunosorbent Assay (ELISA)

The same cell culture plates used in the MTT assay were used to measure secreted interleukin 6 (IL-6) and indoleamin 2,3-dioxygenase (IDO) by ELISA. Cells from all donors (*n* = 8) were used for this experiment. Supernatants from the MTT plates were collected and frozen at −80 °C. IL-6 (Human IL-6 ELISA KIT, Diaclone SAS, Besançon, France) and IDO (human indoleamine 2,3-dioxygenase ELISA Kit, Novus Biologicals, CO, USA) were quantified using “sandwich ELISA”. Samples were thawed at room temperature, centrifuged and diluted 1:10 with the standard diluent buffer from the kit. A total of 100 μL of each of the dilution series (in pg/mL) samples, controls and the blank negative sample (standard diluent) were distributed in duplicate in a 96-well plate pre-coated with IL-6 or IDO capture antibodies. After adding diluted biotinylated anti-IL-6 (50 μL/well), the incubation was carried out for 60 min at room temperature on a shaking plate (type KL2, Edmund Bühler GmbH, Hechingen, Germany). Wells were washed three times with the washing buffer to remove non-specifically bound antigens. Steptavidin–horseradish peroxidase (HRP) solution (100 μL/well) was then applied. After 30 min at room temperature on the shaking plate, three washing steps were performed before adding 100 μL/well of 3,3′,5,5′-tetramethylbenzidine (TMB) substrate solution. The plate was incubated for 10 min in the dark. This resulted in a blue coloration of the solution by HRP. To stop the reaction, 100 μL/well H_2_SO_4_ was added, causing yellow coloration. The color intensity was determined at 450 nm in the ELISA reader and converted into mass/volume. The evaluation was carried out using GraphPad Prism Version 5.01.

### 2.8. Quantitative Real-Time Polymerase Chain Reaction (qRT-PCR)

Gene expression was quantified by qRT-PCR. Cells from all donors (*n* = 8) were used for this experiment. After detaching and counting the cells, 2 × 10^5^ cells/well were seeded in duplicates. Plates were treated with cisplatin concentrations of 5 μM, 10 μM and 20 μM and a negative control with DMEM-EM for 24 h. The Quiagen RNeasy Kit (Qiagen GmbH, Hilden, Germany) was used for RNA isolation. A photometric RNA content measurement was carried out, for which 79 μL DEPC-H_2_O (Sigma-Aldrich Chemie GmbH, Steinheim, Germany) were placed in a UVette (Eppendorf AG, Hamburg, Germany), 1 μL of the RNA solution was added, the content was analyzed in the photometer (Eppendorf AG, Hamburg, Germany) and 80 μL DEPC-H_2_O served as a reference. RNA samples were then frozen at −80 °C until analysis.

To synthesize cDNA from RNA, samples were thawed at room temperature. For each cisplatin concentration, 3 wells were equipped in a reaction vessel (Fast Reaction Tubes, Micro Amp 8 Cap strips, Life Technologies Corp. [Applied Biosystems], Carlsbad, CA, USA). Quantities of 50 ng/well of RNA, 4 μL of Superscript (SuperScript^®^ VILO Mastermix Taq Man Gene Expression Master Mix, Life Technologies Corp. [Applied Biosystems], Carlsbad, CA, USA) and DEPC-H_2_O were pipetted to obtain a volume of 20 μL/well. The vessel was sealed with adhesive foil (Sarstedt AG & Co. KG, Nümbrecht, Germany) and centrifuged at 1300 rpm for 3 min. For cDNA synthesis, the vessel was placed in the qRT-PCR cycler (StepOnePlus Real-Time PCR System, Life Technologies Corp. [Applied Biosystems], Carlsbad, CA, USA), which resulted in a double-stranded cDNA. After the program had been run, the finished cDNA was transferred to eppis and frozen at −80 °C.

TaqMan probes and GAPDH as a reference gene were used (IDO 1, Assay-ID Hs00984148_m1, IL 6, Assay-ID Hs00174131_m1, GAPDH, Assay-ID Hs00985639_g1; all from Life Technologies Corp. (Applied Biosystems), Carlsbad, CA, USA). Three wells were created for each cisplatin concentration per probe on the 96-well plate (Micro Amp Fastoptical 96-Well Plate, Applied Biosystems, life technologies GmbH, Darmstadt, Germany). For each assay, the mixture was prepared in 1.5 mL Eppis, which contained 10 μL master mix, 7 μL DEPC-H_2_O and 1 μL of the corresponding assay per well. Quantities of 18 μL/reaction of the mixture and 2 μL/reaction of the respective cDNA were mixed, and the plate provided with an adhesive film, centrifuged at 860 rpm for 5 min and placed in the qRT-PCR cycler. After incubation at 95 °C for 10 min, 40 replication cycles were run and, after completion, reached the CT value for each sample, from which the relative content (fold change) could be calculated.

### 2.9. Illustration of Data and Statistical Evaluation

GraphPad Prism version 5.01 was used to create the graphs and for statistical analysis. The paired *t*-test was used for the evaluation of normally distributed measured values. Values not normally distributed were determined using Wilcoxon’s test, or Friedmann’s and Dunn’s tests for more than paired groups. A *p*-value of *p* ≥ 0.05 was set as the level of significance (marked with “*”).

## 3. Results

### 3.1. Characterization of Mesenchymal Stromal Cells

**Figure 1:**
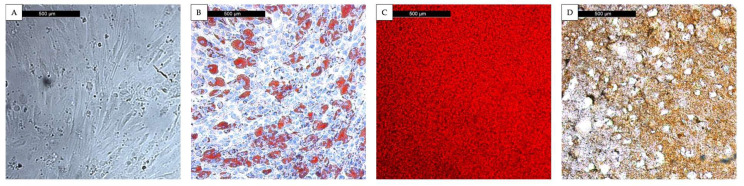
Staining after differentiation confirmed the presence of adipogenic and osteogenic phenotypes in MSCs from two donors. Donors 3 and 6 were included (*n* = 2), (**A**) MSC native in DMEM-EM; (**B**) red fat vacuoles in the Oil Red O staining (adipogenic differentiation); (**C**) red–orange calcium deposits in the Alizarin Red S staining and (**D**) brown–black calcium deposits in the Von Kossa staining (osteogenic differentiation).

In order to test the identity of cells isolated and treated in medium for differentiation into an MSC phenotype (specific osteogenic, chondrogenic and adipogenic phenotypes), cells were stained using three different methods. The presence of appropriate adipogenic and osteogenic differentiation was evaluated (Figure 1). Stainings confirmed the presence of adipogenic and osteogenic phenotypes.

**Figure 2:**
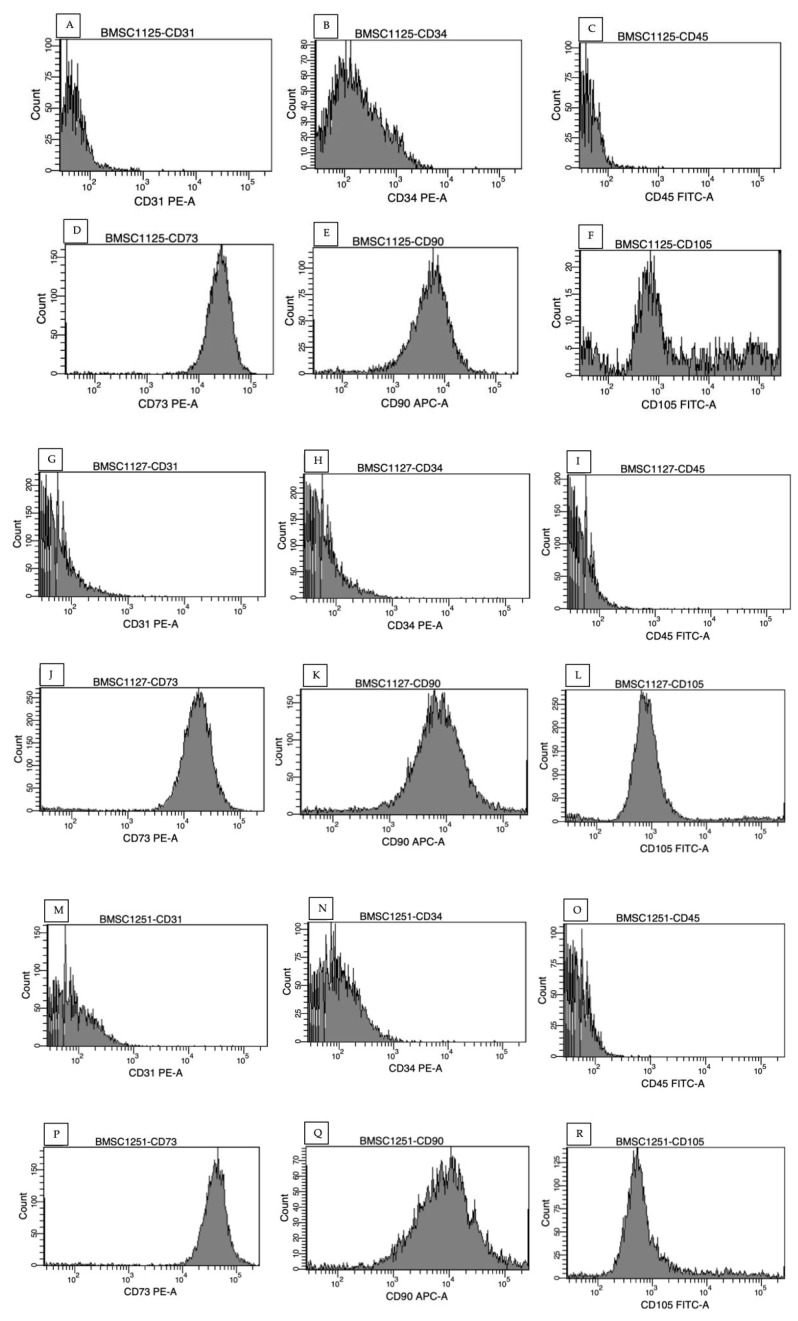
MSC-typical constellation of six surface antigens was evaluated by flow cytometry. Here, the constellation of three donors is shown. Donors 4, 5 and 6 were included (n = 3), negativity for CD31 (**A**,**G**,**M**), CD34 (**B**,**H**,**N**), CD45 (**C**,**I**,**O**), positivity for CD73 (**D**,**J**,**P**), CD90 (**E**,**K**,**Q**), CD105 (**F**,**L**,**R**).

Cells were further examined for the stromal cell-typical constellation of surface antigens using flow cytometry. Surface antigens CD73, CD90 and CD105 were detected, whereas CD31, CD34 and CD45 were not detected on the surface of cells. These results were reproducible for three donors, as shown in Figure 2.

### 3.2. Toxicity of Cisplatin in the MTT Assay

**Figure 3:**
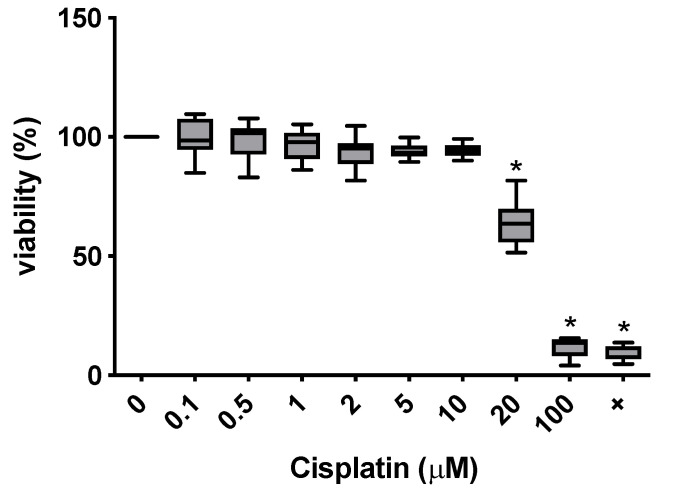
Concentration-dependent cytotoxicity of cisplatin on MSC in the MTT assay, as measured by relative viability compared to the negative control (0 μM). A significant reduction in viability was detected from 20 μM and also in the positive control. Asterisks indicate statistically significant results.

After establishing the MSC characteristics of isolated cells, subtoxic cisplatin concentrations were sought in order to determine appropriate conditions to evaluate immune modulatory effects of cisplatin on MSCs at those concentrations. MSCs from four donors were treated with increasing concentrations of cisplatin (0.1 μM, 0.5 μM, 1 μM, 2 μM, 5 μM, 10 μM, 20 μM and 100 μM) and viability was determined. Cisplatin showed a cytotoxic effect on MSCs at 20 µM, with a sub-toxic concentration range up to 10 µM. At these concentrations, the viability of MSCs remained stable in all donors tested, indicating that cell integrity and metabolic function is maintained under these conditions (Figure 3).

**Figure 4:**
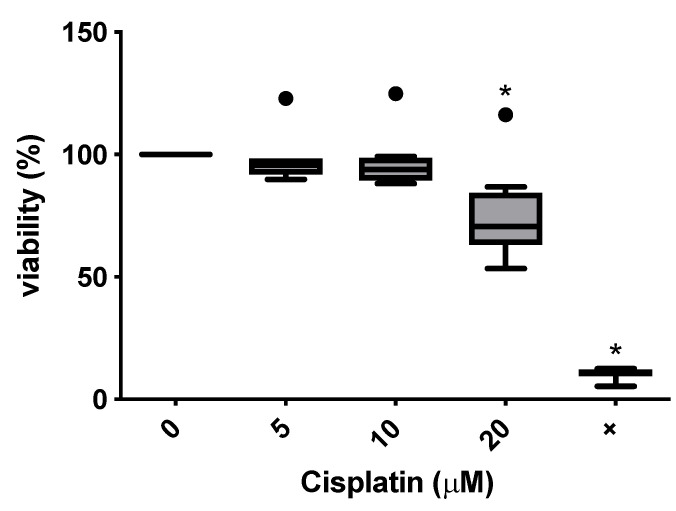
Concentration-dependent cytotoxicity of cisplatin on MSCs using doses of 5 μM, 10 μM and 20 μM. Donors 1 to 8 were included (*n* = 8). Asterisks indicate statistically significant results. Bullets indicate outliers.

In order to represent the subtoxic range and the transition range from subtoxic to toxic, further experiments were carried out with cisplatin concentrations of 5 μM, 10 μM and 20 μM, as well as a negative control (medium only) (Figure 4). The test was repeated in this range using cells from eight donors.

The onset of cytotoxicity was reproducibly detected at 20 μM cisplatin, which was relevant for the concentrations used for further immunomodulatory experiments.

### 3.3. Immunomodulatory Effects on Protein and mRNA Levels

It was thoroughly demonstrated that isolated MSCs from 8 donors remain >95% viable after treatment with up to 10 µM cisplatin. Since MSCs play a crucial role in the tumor microenvironment, and cisplatin is a common therapy for several cancers, the immunomodulatory effects of cisplatin on MSCs were investigated. Concentrations of mRNA and protein for the tumor cell promotor and inflammation mediator IL-6 and immunosuppressor and tumor promotor IDO1 were determined in MSC medium supernatants with and without cisplatin treatment.

**Figure 5:**
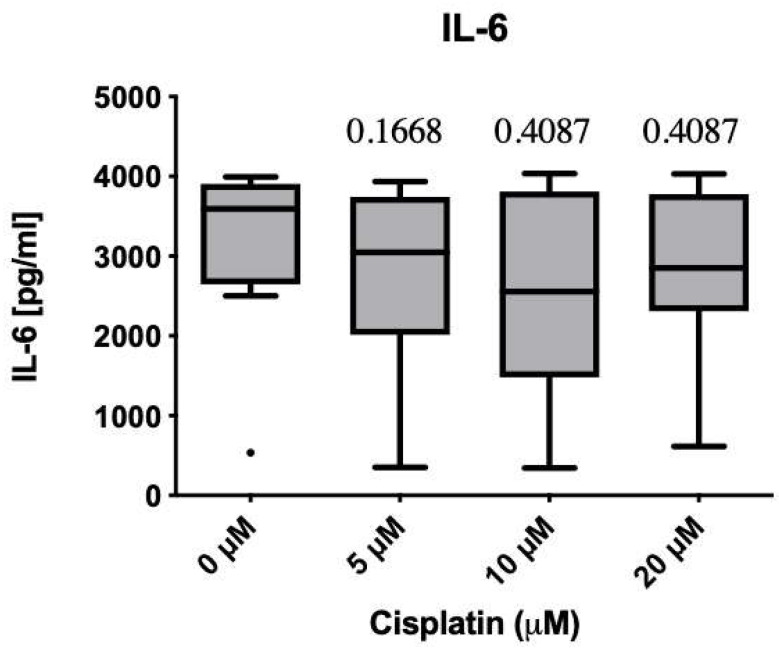
ELISA for IL-6, donors 1 to 8 were included (*n* = 8). Concentration-dependent decrease within the subtoxic range. Weaker median effect at 20 μM. The effect does not reach the level of significance. *p*-values for statistical significance compared to the control group indicated directly above the bars. Bullet indicates outlier.

**Figure 6:**
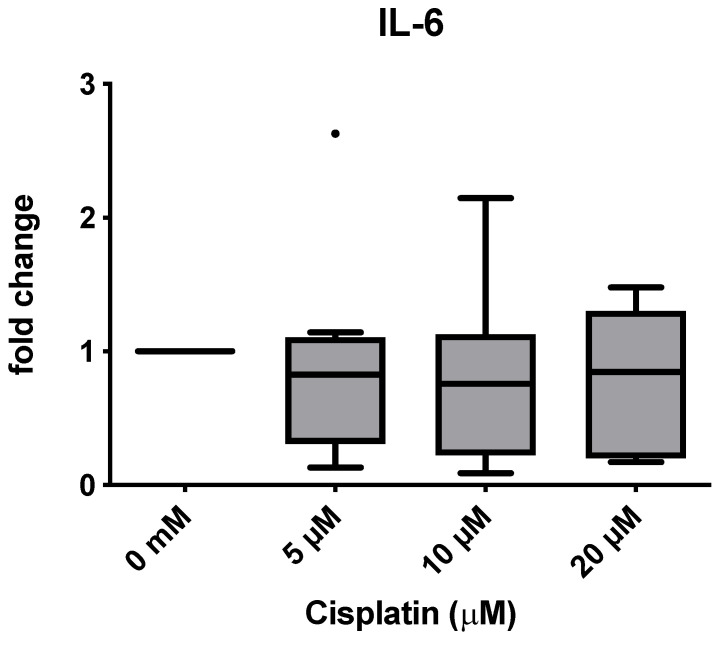
Decrease in IL-6 expression in qRT-PCR without reaching the level of significance. Donors 1 to 8 were included (*n* = 8). Bullet indicates outlier.

IL-6 median protein level qualitatively decreased after cisplatin treatment (Shapiro–Wilk normality test, *p*-values: 5 µM = 0.1668; 10 µM = 0.4087; 20 µM = 0.4087) with approximately 1000 pg/mL at the most. While the median protein level decreased steadily with increasing cisplatin concentration (0 µM, 5 µM and 10 µM), this trend did not continue at the slightly toxic concentration of 20 μM, although the median remained below the untreated group (Figure 5). A pronounced donor variance could be seen, and in some cases, the IL-6 concentration was more than halved. The strongest decrease was observed at 10 µM. However, this trend did not reach statistical significance, which is also reflected in the mRNA expression values. Here, too, a certain decrease was noticeable, although the statistical significance level was not reached (Figure 6).

**Figure 7:**
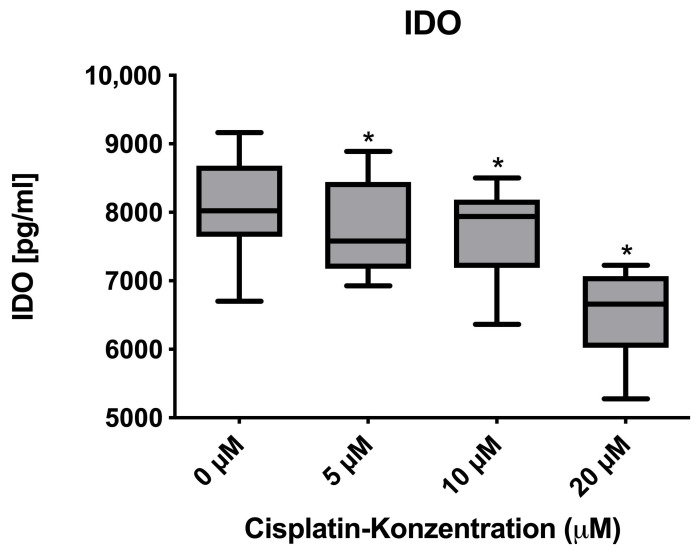
Concentration-dependent significant decrease in IDO in the ELISA. Donors 1 to 8 were included (*n* = 8). Asterisks indicate statistically significant results.

**Figure 8:**
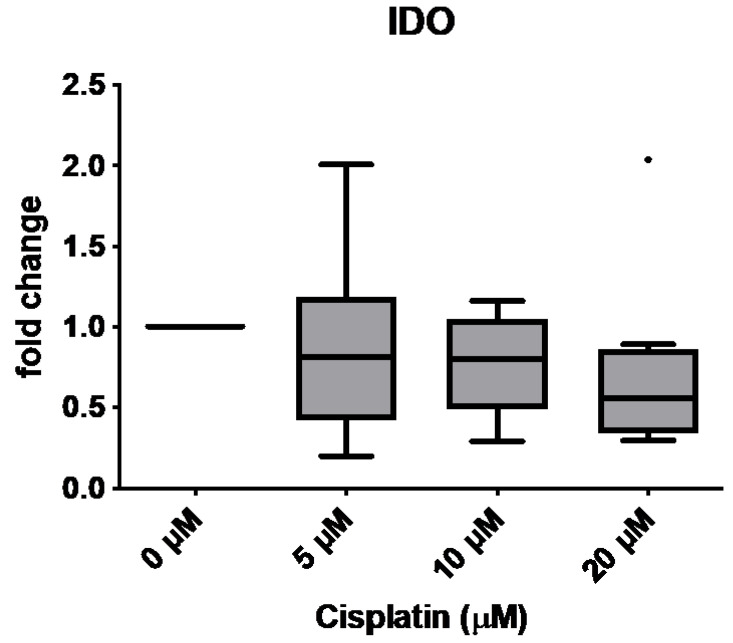
Decrease in the expression of IDO in the qRT-PCR without reaching the level of significance. Donors 1 to 8 were included (*n* = 8). Bullet indicates outlier.

IDO1 protein levels significantly decreased in a cisplatin concentration-dependent manner (Figure 7; paired *t*-tests, *p*-values: 5 µM = 0.0028; 10 µM = 0.0023; 20 µM = 0.0058, all concentrations compared to negative control). This decrease in protein expression was approximately the same at all tested concentrations compared to the untreated control. Slight differences were detected for IDO1 at the mRNA-level; this effect was not statistically significant (Figure 8).

### 3.4. Migration Analysis Using Scratch Assay

**Figure 9:**
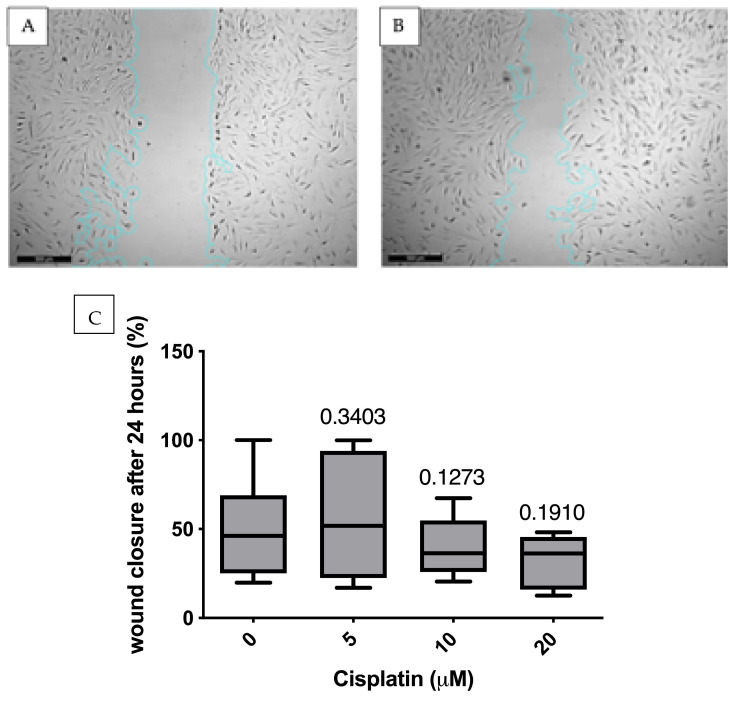
(**A**,**B**) Migration progress in the scratch assay (here without cisplatin treatment) at 0 (**A**) and 24 h (**B**). 0 h: straight after adding the cell-free aisle. 24 h: narrowing of the aisle as a sign of wound closure. (**C**) no significant change in migration after treatment with cisplatin (*t*-Tests). *p*-values for statistical significance compared to the control group indicated directly above each boxplot.

Minor molecular changes were detected. In order to tap into the relevance of those results, a functional test, the scratch assay, was applied. Here, the ability of MSCs to migrate after treatment with cisplatin was analyzed in the scratch assay. Here, the speed at which an introduced cell-free lane (scratch) “closes” as a model for a wound is considered. The percentage of wound closure, relative to time 0, was analyzed at 24 h. There was no statistically significant influence on migration after cisplatin treatment (Figure 9), although the morphology of the lane appeared to differ qualitatively. Although cisplatin affects some functions of MSCs, migration ability is not affected under the conditions tested.

## 4. Discussion

**Figure 10:**
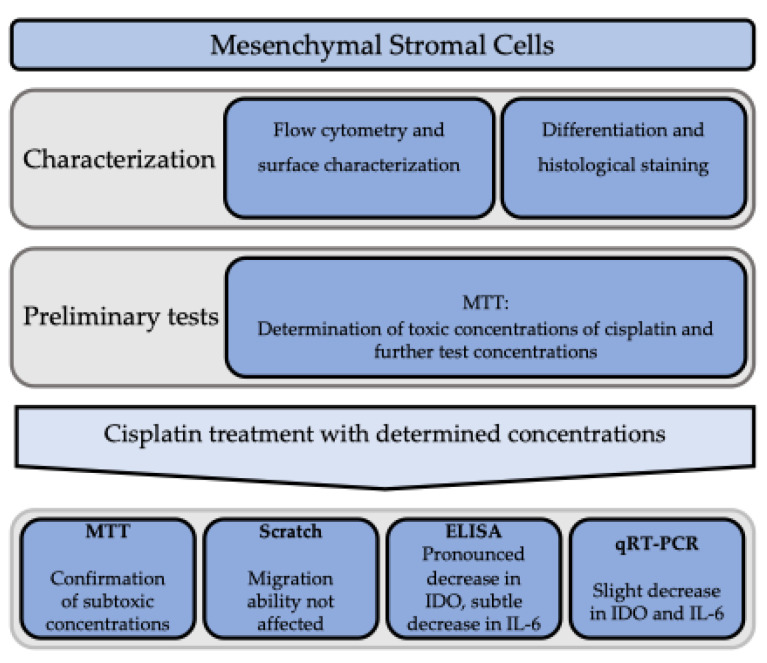
Illustration of the experiments conducted and the investigated effects of cisplatin on MSCs and the expression of IL-6 and IDO.

The aim of this work was to evaluate the immunomodulatory effects of cisplatin on human MSCs. The widespread use of cisplatin in tumor therapy, combined with the important role of MSCs in the tumor microenvironment, motivated the authors to investigate molecular details of potential immunomodulation of MSCs by cisplatin, and thereby effects on tumor development. Specifically, the focus was placed on the immunomodulators and tumor promotors IL-6 and IDO1. For the MSCs used, the common criteria for their identification were demonstrated. In the present work, cisplatin showed a concentration-dependent cytotoxicity in MSCs. A significant reduction in viability occurred from doses of 20 μM cisplatin. The MSCs proved to be relatively stable. This observation is consistent with observations from the literature, since MSCs have previously been shown to be relatively resistant, including to cisplatin-induced cytotoxicity [25]. As expected, subcytotoxic doses of cisplatin did not cause major effects in terms of impairment of migration ability (findings summarized in Figure 10).

The current study, hence, adds details to this established understanding, suggesting that immunomodulation via IL-6 and IDO1 of the tumor microenvironment by MSCs during cisplatin treatment, even at subtoxic doses, is a relevant mechanism to consider. The decrease in protein expression for IDO1 and IL-6 under subtoxic concentrations of cisplatin could be seen in each case, whereby only the results for IDO1 reached statistical significance. In contrast, the changes in mRNA levels in qRT-PCR of both IDO1 and IL-6, while a congruent reduction is also visible, hardly reached statistical significance. Superficially, this suggests post-transcriptional regulation or a dissociation of mRNa and protein regulation under cisplatin treatment in the current setting. However, mRNA and protein stability, translation efficiency and feedback loops are examples of parameters which may obscure regulatory effects. For a deeper understanding, such factors must be investigated and taken into consideration.

Several mechanisms of action of the antitumor immunomodulation of cisplatin are generally assumed in the literature: upregulation of MHC class I and the lytic activity of cytotoxic effectors, recruitment and proliferation of effector cells and downregulation of the immunosuppressive microenvironment [20]. The immunomodulatory effects also give hope for possible relevant synergistic effects with other tumor-active substances, such as paclitaxel [26,27], ifosfamide [27], p53 cancer vaccine [28], vinorelbine [29] and 5-fluorouracil (5-FU) [30]. With regard to the downregulation of the microenvironment, a distinction can be made between anti- and pro-tumor immune effects. A reduction in myeloid-derived suppressor cells (MDSC) by cisplatin has, for instance, been demonstrated in both animal experiments and in the tumor-suppressive microenvironment [26,31,32]. These studies, and partly contradictory data, underline the complexity of tumor biology and demonstrate the importance of paying attention to detail and exact conditions in studies, in vivo as well as in vitro, in order to elucidate the exact parameters, which induce anti- or pro-tumor effects.

Self-renewing multipotent MSCs have a regulatory function in the field of tumor biology and anti-tumor immunity. In particular, they influence chemokine and cytokine production [11,12,13], change the proliferation of CD8+ T lymphocytes [18] and natural killer cells [33,34] and regulate the formation of myeloid suppressor cells [35], regulatory T cells [36] and the antigen-presenting properties of dendritic cells [37]. However, they are themselves influenced by the cytokine profile of their environment, such as in the tumor microenvironment, wherein tumor-infiltrating immune cells may induce pro- or anti-tumor effects through an interplay between MSCs and the immune system. Often, the mechanisms behind the effects described above are poorly understood. A better understanding of these mechanisms is an important component for exploiting therapeutic potentials in a possible clinical implementation of combined MSC and drug-based tumor therapy [12,38,39]. This paper addresses two well-known tumor microenvironment modulators in the context of cisplatin-treated MSCs: IDO1 and IL-6.

For the first time, we were able to demonstrate a reduced IDO1 protein expression through the application of subtoxic concentrations of cisplatin to isolated MSCs in vitro.

IDO1 is immunosuppressive and tumor-stimulating and is secreted, among others, by MSCs. In two humanized mouse models for melanoma and lymphoma with added MSCs, IDO1 inhibited the proliferation of CD8(+) T cells and B cells and promoted tumor growth. The effect was reversible with the IDO1 inhibitor 1-methyl tryptophan (1-MT) [18]. It has also been suggested that IDO could play a crucial role in tumor immune escape. Thus, inhibiting IDO expression in the tumor microenvironment was proposed as a strategy for addressing tumor immune tolerance [40,41]. It was assumed that there was increased IDO expression by cells recruited in the tumor microenvironment due to soluble factors secreted by the tumor [42]. The current results shed some light on this, suggesting MSCs as a drug-targetable source of IDO1.

In addition to IDO1, IL-6 protein levels qualitatively decreased upon cisplatin treatment. A possible impact of cisplatin on MSC-derived IL-6 has, to the best of our knowledge, not been reported previously. As a proinflammatory cytokine, IL-6 has physiological functions in response to infections and injuries, by stimulating acute phase responses, and hematopoiesis [43,44]. IL-6 from MSCs has been shown to induce differentiation and activation of osteoclasts via the RANK ligand (RANKL) [45], which is associated with bone resorption and osteoporosis. IL-6-promoted excessive induction of the vascular endothelial growth factor (VEGF) has been demonstrated, which leads to angiogenesis and vascular permeability and represents pathological mechanisms in inflammatory processes [46]. Clinical studies have demonstrated that anti-angiogenic, VEGF-targeting drugs have beneficial anti-tumor effects, but these were of a transient nature and were followed by tumor recurrence. Given the above-described IL-6 induction of VEGF, one could hypothesize better anti-tumor effects by the simultaneous inhibition of IL-6 and VEGF might prolong such anti-tumor effects. There is some speculation that bone marrow-derived cells (BMDCs), along with stromal cells, play an important role in the development of resistance to those anti-VEGF therapies, highlighting the importance of studying the tumor microenvironment when new therapies are considered. It has also been shown that the development of resistance is partly mediated by proangiogenic cytokines and growth factors, including IL-6 [47]. The relevance of the connection between inflammation and tumors is also reflected by high IL-6 levels in the tumor microenvironment [48,49]. Specifically, malignant tumors benefit from its pronounced pro-tumor properties via, e.g., improved tumor survival, proliferation, angiogenesis, invasiveness and metastasis [50,51] and by protecting the tumor through antioxidant and antiapoptotic signaling pathways [52,53,54]. Therefore, IL-6 inhibition is considered a possible tumor therapeutic strategy [55].

Some shortcomings of our study should not remain unmentioned. The presented results were obtained under in vitro conditions, and thus direct transfer to the complex processes in vivo was not possible. In addition, not all MSCs used were individually tested for the usual MSC criteria, which is due to the limited primary cell material from donors. For example, the number of donors in the staining to detect MSC differentiation was only two. In qRT-PCR, a higher variance is visible for IL-6 at 10 uM cisplatin and for IDO at 5 uM cisplatin than for the other concentrations. This can be explained by outliers in the measurements, which can be caused, for example, by donor variance, since primary human donor MSC were used. Of course, certain deviations can also arise from technical issues during experimental work.

Nevertheless, the current study has contributed baseline data of isolated MSCs treated with cisplatin, without the influence of factors secreted by tumor cells. It could be shown that IL-6- and IDO1-dependent immunomodulation by MSCs in the tumor microenvironment could play a role during cisplatin treatment. The decrease in protein expression for IDO and IL-6 was detectable under subtoxic concentrations of cisplatin in all cases. Such data demonstrate a cellular proof-of-concept for the interaction between cisplatin and MSCs, upon which more complex studies containing, e.g., additional immune components or tumor cells, may be designed. The minor mRNA downregulation in qRT-PCR, despite protein suppression, may indicate post-transcriptional regulation as a major regulatory mechanism, which justifies further investigation of the mechanism of action of cisplatin at the translational level. What should also be noted is that, during in vivo tumor therapy, treatment is not subtoxic but toxic, which also affects the TME. It would be interesting to repeat subtoxic doses several times and then observe the effects, particularly assessing whether the repetitive doses affect long-term cytokine release differently.

## 5. Conclusions

The results of this study suggest that, in addition to previously known effects, cisplatin may also act by a novel mechanism: suppressing immunomodulatory proteins IL-6 and IDO1 in MSCs. This has not previously been described for MSCs. Such an influence could have an impact on the tumor microenvironment during cisplatin therapy, and thus on overall tumor biology. Possibly, this mechanism contributes to the success of cisplatin as one of the most important chemotherapeutic agents, for example, in the context of combination therapies. Extended studies on this topic are therefore of great interest. Also, the investigation of other cytokines, such as IL-8 or TGF-ß, would be relevant to further explore these hypotheses. Additionally, the MSCs’ immunomodulatory effect outside the tumor microenvironment, particularly in the context of chemotherapy, warrants further investigation. This, in turn, could shed light on the undesirable side effects of cisplatin. In vivo studies to determine the translational impact of the effects described here are required to develop future novel therapeutic concepts. Taken together, this study suggests previously undescribed subtle immunomodulatory effects of cisplatin which provide a baseline for mechanistic investigations into MSCs’ role in the modulation of the tumor microenvironment and beyond.

## Figures and Tables

**Table 1:** Antibodies used for surface characterization.

Antibody	Source
APC mouse anti-human CD90 (559689)	Becton Dickinson GmbH, Heidelberg, Germany
FITC mouse anti-human CD105 (555690)	Becton Dickinson GmbH, Heidelberg, Germany
FITC mouse anti-human CD45 (555482)	Becton Dickinson GmbH, Heidelberg, Germany
PE mouse anti-human CD31 (555446)	Becton Dickinson GmbH, Heidelberg, Germany
PE mouse anti-human CD34 (550761)	Becton Dickinson GmbH, Heidelberg, Germany
PE mouse anti-human CD73 (550257)	Becton Dickinson GmbH, Heidelberg, Germany
FITC, Alexa Fluor^®^ 488 goat anti-mouse IgG, 1:1000	Life Technologies Corporation, Carlsbad, CA, USA)

## Data Availability

Data are contained within the article.
